# Machine Learning Photovoltaic String Analyzer

**DOI:** 10.3390/e22020205

**Published:** 2020-02-11

**Authors:** Sandy Rodrigues, Gerhard Mütter, Helena Geirinhas Ramos, F. Morgado-Dias

**Affiliations:** 1 Instituto de Telecomunicacoes of the Instituto Superior Tecnico of the University of Lisbon, 1049-001 Lisbon, Portugal; hgramos@ist.utl.pt; 2 Laboratory for Robotics and Systems in Engineering (LARSyS), Madeira Interactive Technologies (M-ITI) and Institute and Interactive Technologies Institute (ITI), 9020-105 Funchal, Portugal; 3 ALTESO GmbH, 1010 Vienna, Austria; muetter@alteso.at; 4 Faculty of Exact Sciences and Engineering, University of Madeira, 9020-105 Funchal, Portugal; morgado@uma.pt

**Keywords:** machine learning prediction models, PV string, PV fault, hybrid methodology, ensemble methodology

## Abstract

Photovoltaic (PV) system energy production is non-linear because it is influenced by the random nature of weather conditions. The use of machine learning techniques to model the PV system energy production is recommended since there is no known way to deal well with non-linear data. In order to detect PV system faults, the machine learning models should provide accurate outputs. The aim of this work is to accurately predict the DC energy of six PV strings of a utility-scale PV system and to accurately detect PV string faults by benchmarking the results of four machine learning methodologies known to improve the accuracy of the machine learning models, such as the data mining methodology, machine learning technique benchmarking methodology, hybrid methodology, and the ensemble methodology. A new hybrid methodology is proposed in this work which combines the use of a fuzzy system and the use of a machine learning system containing five different trained machine learning models, such as the regression tree, artificial neural networks, multi-gene genetic programming, Gaussian process, and support vector machines for regression. The results showed that the hybrid methodology provided the most accurate machine learning predictions of the PV string DC energy, and consequently the PV string fault detection is successful.

## 1. Introduction

Photovoltaic (PV) systems are composed of many electrical components that are prone to faults when exposed to weather conditions. The continuous monitoring of the PV systems ensures their maximum energy production output since the anomalies can be detected and dealt with as soon as possible which contributes to maintaining the return of investment (ROI) payback periods. The amount of energy produced by a PV system depends mainly on the amount of sunlight that it absorbs and is also influenced by other weather components such as the ambient temperature. The PV system energy production is non-linear, since the current that runs in the PV cells and voltage at the PV cell terminals depend on the weather that they are exposed to [1,2,3].

As a result, the use of machine learning techniques to model the behavior of PV system production is recommended for analyzing the performance of PV systems and detecting faults since they are known to deal well with non-linear data [4,5,6]. These machine learning models are data-driven and therefore require historical data measured at nearby weather stations (inputs) as well as from the PV system (outputs). The machine learning modelling process consists of a training stage and a testing stage where both stages require historical data. The historical data used for the training dataset should be measured when the PV system is working well, in order for the trained machine learning models to mimic a PV system that works correctly.

One of the ways to detect PV system faults is to analyze the deviation between the measured data of the PV system production and the estimated/predicted values of the PV system production provided by machine learning regression models as described by Gigoni et al. in [5] and by Jiang et al. in [6].

The accuracy of the PV system fault detection depends on the accuracy of the prediction values provided by the machine learning regression models. Consequently, the accuracy of the PV system production machine learning predictions depends on the type of data that is selected for use in the training stage (training dataset) of the machine learning regression models as well as the type of machine learning technique that is used for modelling [4,5].

There are many techniques that have been used by the research community to select the correct data for a training dataset such as classifying the historical data into “similar days” that include sunny days, cloudy days and rainy days as described in [7]. Another training data selection technique includes subdividing the data according to the seasons of the year, when annual historical data are available as described in [8]. However, the type of training data selection technique that is adopted depends on what historical data are available.

For this work, six PV strings that belong to the same combiner box of a utility-scale ground-mounted PV plant were provided by a green technology company called ALTESO GmbH located in Vienna Austria. The available historical data includes daily solar irradiation measurements in W/m^2^ used as the inputs and daily DC energy measurements in kWh of each of the six PV strings used as the outputs. The data are only from the month of July (31 daily samples) of four different years, namely 2014, 2015, 2016, and 2017. Each season of the year is known to have a different trend in the weather conditions, even though the trend is similar between spring and summer as well as between autumn and winter as described in [7]. For this reason, it was decided that data only from the month of July would be enough to carry out the research experiments of this work in order to minimize the issues caused by the seasonal trend, and therefore data from other months was not requested from ALTESO GmbH.

As described before, the types of machine learning techniques that are used to predict the PV system energy also influence the accuracy of the machine learning regression model prediction outputs. Therefore, five machine learning techniques were chosen for this work, namely: (a) regression tree, (b) artificial neural network (ANN), (c) multi-gene genetic programming (MG-genetic programming), (d) Gaussian process, and (e) support vector machine for regression (SVR). The five machine learning techniques are compared to each other by means of an error analysis by using the root mean squared error (RMSE) and normalized root mean squared error (NRMSE) evaluation metrics.

The accuracy improvement of the machine learning models is a very important issue to address, in order for the data-driven models to mimic the physical system (in this case it is a PV system) as accurately as possible. As a result, there are many research experiments that propose the implementation of new machine learning methodologies designed to improve the machine learning models. All these research experiments that improve the accuracy of the machine learning models can be organized into four main machine learning methodologies such as the data mining methodology, the machine learning technique benchmarking methodology, the hybrid methodology and the ensemble methodology. 

The data mining methodology considers all the techniques that are used to organize the historical data so that the machine learning model outputs are improved, and this includes the training data selection techniques and feature selection techniques. 

The machine learning technique benchmarking methodology considers the comparison of various machine learning models provided by different machine learning techniques in order to determine which one provides the most accurate results. The five machine learning techniques that were selected to be studied in this work will be used in the machine learning technique benchmarking methodology.

The hybrid methodology considers the combination of various modelling sub-fields of the artificial intelligence field such as the combination of a fuzzy logic modelling system and a machine learning modelling system. Other hybrid methodologies include the combination of a physical model and a machine learning model to provide the most accurate machine learning results such as the PHANN [9]. 

The ensemble methodology considers the averaging of all the results of various machine learning models provided by different machine learning techniques and also provided by variations of the same machine learning technique (decision trees) such as the random forests [10].

This information about the different methodologies served as motivation to devise various experiments that would test out all of these machine learning methodologies in order to determine which one would provide the most accurate machine learning model outputs of the daily DC energy of each of the six PV strings. As a result, six experiments and 41 scenarios that are fully described in the methodology Section were devised. Experiments 1 and 2 consider both the data mining methodology (training data selection) as well as the machine learning benchmarking methodology (benchmark the five machine learning techniques). 

A new hybrid methodology is proposed in this work and is described in Experiment 3. This new hybrid methodology is designed to improve the machine learning model prediction accuracy which combines the use of a fuzzy system and the use of five trained machine learning models. 

A new ensemble methodology is also proposed in this work, which averages the results of the five trained machine learning models by using a simple mean equation. Experiments 4, 5, and 6 consider the ensemble methodology where each of the experiments includes an ensemble technique (averaging method) that is configured differently. The ensemble technique of Experiment 4 averages all the results of the five machine learning techniques. The ensemble technique of Experiment 5 averages the top three results of the five machine learning techniques. Finally, the ensemble technique of Experiment 6 averages only the results of the less popular machine learning techniques such as the regression tree, multi-gene genetic programming and the Gaussian process (the ANN and SVR are referred to as the more popular machine learning techniques). 

The aim of this work is to accurately predict the daily DC energy output of the PV strings of a utility-scale PV system by using a data-driven machine learning approach. In order to achieve this aim, the results of four machine learning methodologies known to improve the accuracy of the machine learning models were benchmarked. The four machine learning methodologies are namely the data mining methodology, machine learning technique benchmarking methodology, hybrid methodology, and the ensemble methodology. The machine learning PV string performance analyzer proposed in this work has the task of detecting anomalies in the solar production output of the PV strings in order for the maintenance team to act on them as soon as possible and to ensure maximum PV system output efficiency. This task is usually carried out by performing a deviation analysis between the measured data from the PV plant strings and the estimated data provided by the machine learning technique prediction models of the corresponding PV plant strings [5,6]. However, the results of this work also show that the comparison of the prediction values (provided by the machine learning regression models) between the neighboring PV strings can also be used to detect PV string anomalies.

The organization of this work is as follows: after the introduction all the experiments and scenarios devised in this work are described in the methodology, Section 2. The results of the experiments are discussed in the results and discussion, Section 3, and finally the main conclusions are mentioned in the conclusion, Section 4.

## 2. Materials and Methods 

This section describes the six experiments that were used to determine which machine learning methodology should be implemented to provide the most accurate machine learning regression model outputs of the daily DC energy of the PV system strings. 

The historical data associated with PV systems include weather data and PV system data. The weather data are measured by on-site or remote weather stations that measure the solar irradiation, ambient temperature, wind speed etc. while the PV system data are measured by PV monitoring systems that measure the power and/or energy produced by the PV system both on the DC (direct current) or/and the AC (alternate current) side of the PV system. An inverter divides the PV system into the AC and DC side, since it is used to invert the DC current produced by the PV modules into the AC current which is used in the electrical grid, and therefore the DC side of the PV system refers to the components between the PV modules and the inverter while the AC side of the PV system refers to the components between the inverter and the electrical grid. The machine learning techniques provide a model based on the relationship between the weather data (inputs) and the PV system data (outputs) of the historical data.

For the scope of this work, the historical data from six PV strings that belong to the same combiner box and the historical on-site solar irradiation measurements were selected and they includes daily samples from the month of July of four consecutive years namely the years 2014, 2015, 2016 and 2017. Since the measurements of the PV strings are made in the combiner box the PV system data consists of DC energy measurements. Each PV string is associated with 31 daily input samples (solar irradiation) and 31 daily output samples (DC energy measurements), since the measurements were made during the 31 days of the month of July. Therefore, the total number of samples that are available is 744, associated to the data measured from the six PV strings in four years (6 PV strings x 31 July days x 4 years).

The machine learning modelling process consists of various procedures such as

1. Acquiring and pre-processing the historical data
2. Selecting the machine learning techniques
3. Training and testing the machine learning techniques
4. Analyzing the model error

In this work, the pre-processing of the raw historical data consists of normalizing the historical data samples and dividing the PV string samples into two groups. The raw historical data are pre-processed before training the prediction models, by normalizing both the inputs and outputs with a pre-processing technique called *Min-Max* normalization to speed up the prediction model training process. The minimum and maximum values of the input samples (accumulated solar irradiation values) were respectively 1 W/m^2^ and 10000 W/m^2^, while the minimum and maximum values of the output samples (accumulated PV system DC energy values) were respectively 1 kWh and 40 kWh. These values are the highest and lowest values that the PV string experienced during the month of July over all four years. 

There is a vast number of machine learning techniques to choose from, however the most popular ones among the research community are namely the artificial neural networks (ANN) and the support vector machines (SVM). In order to verify which machine learning technique provides the most accurate model results for the problem at hand, a benchmark analysis is performed by evaluating the models provided by various machine learning techniques as described in [7].

The vast number of machine learning techniques can be narrowed down by following the concept introduced by Domingos in his book [11], where he suggests that all machine learning techniques can be organized into five “tribes”, in which each one is represented by its own master algorithm (Table 1). This machine learning selection scheme represents all types of machine learning techniques from the machine learning field of research. Based on this information, five machine learning techniques were chosen for this work and are namely: the (a) regression tree, (b) artificial neural network, (c) multi-gene genetic programming, (d) Gaussian process, and (e) support vector machine for regression.

The training and testing steps of the machine learning modelling process require their own sets of historical data which are referred to as the training dataset and testing dataset. These two datasets should contain different data in order to correctly evaluate the model. The type of historical data selected for the training dataset influences how well the model generalizes to new unseen data (testing dataset) when the testing procedure takes place. For the scope of this work, the 2015 July PV string data are used for the training dataset, while the July PV string data of years 2014, 2016, and 2017 are used for the testing dataset. 

The last procedure of the machine learning modelling process is to perform an error analysis of the machine learning model by using evaluation metrics to calculate the error of the machine learning model. The evaluation metrics used in this work are related to the machine learning regression models that are used to predict the PV string energy and they include the root mean squared error (RMSE) and the normalized root mean squared error (NRMSE). The RMSE is easily interpreted due to having the same units as the sample outputs (kWh) and calculates the average error compared to the measured output value (Equation (1)). A large positive RMSE value represents a large deviation scale in the prediction values from the measured values, and therefore represents a large error value, and therefore the closer to zero the RMSE value is the better. As previously described, the maximum amount of daily DC energy produced by the PV strings during the month of July in any of the four years is 40 kWh, and therefore the RMSE and NRMSE error values are related and compared to this maximum energy value. The NRMSE provides the average percentage value of the error (Equation (2)), in relation to the difference between the unnormalized minimum and maximum output values, which is 39 kWh (40 kWh – 1 kWh = 39 kWh) assumed in this work, where *Yt* is the measured value and *Yp* is the predicted output and n is the number of observations. Therefore, a 5% NRMSE value is assumed as acceptable for the scope of this work since it represents a 1.95 kWh RMSE value.

(1) $$RMSE=\;\sqrt{\frac{1}{n}\overset{n}{\underset{i=1}{\sum }}{\left(Yt-Yp\right)}^{2}}\;$$

(2) $$NRMSE=\;\frac{RMSE}{Y_{tmax}\;-\;Y_{tmin}}$$

One of the main goals of the six research experiments and 41 scenarios that were devised is to provide information about the types of machine learning techniques that should be selected to provide the most accurate model results. The other main goal of these experiments and scenarios is to provide information about the machine learning methodology that should be implemented to provide the most accurate model results. 

**Figure 1:**
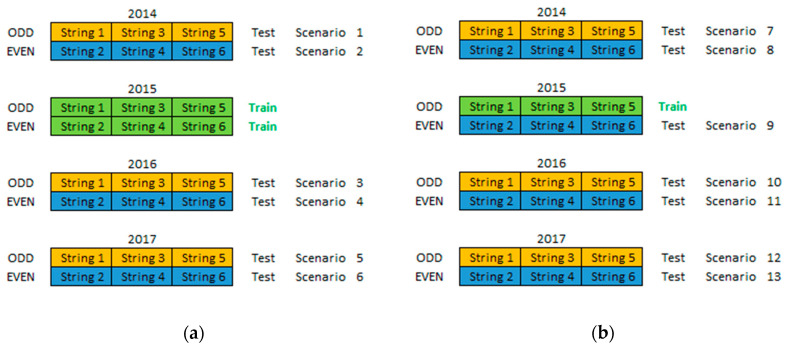
Illustrating the Training and Testing Datasets of (**a**) Experiments 1 and (**b**) Experiment 2.

As described before, one of the most important procedures to take place is the selection of the samples to include in the training dataset. This training data selection process is part of the machine learning data mining methodology that is used in this work, as described in the introduction Section. In order to understand what number and what types of samples to include in the training dataset for the machine learning regression model to generalize well to new unseen data (testing dataset) and provide the most accurate prediction outputs, Experiments 1 and 2 were devised as illustrated in Figure 1. All the historical data are divided into two groups namely the “ODD” strings (orange) and the “EVEN” strings (blue) and each of these groups is associated with a year and a dataset (training or testing). In Experiment 1 the 2015 “ODD” and “EVEN” PV string historical data (6 PV strings with 186 samples) were used for the training dataset (green) while all the other historical data were used for the testing dataset. In Experiment 2 only the 2015 “ODD” PV string historical data (3 PV strings with 93 samples) were used for the training dataset, while all the other historical data were used for the testing dataset. This dataset size (93 samples) has been proven to be adequate to provide accurate machine learning model results as described in [7] and in [12].

At the same time these two experiments are performing the machine learning data mining methodology to determine which historical data should be included in the training dataset, they are also performing the machine learning technique benchmarking methodology described in the introduction section since the five machine learning techniques are used in the training process. Therefore, the comparison between these two experiments provides information about what number of samples needs to be included in the training dataset to train the machine learning regression model as well as information about which machine learning technique is able to provide the most accurate results.

The Machine Learning for Regression model parameter settings for this work are mainly the Matlab^®^ 2017b default Machine Learning model settings. The machine learning techniques were only trained with simple/default parameter settings and then tested. Validation and cross-validation were not performed on any of the models in this work unless automatically done by the fitting command of the Matlab^®^ 2017b toolbox. 

Experiment 3 considers the hybrid methodology where a Fuzzy Logic Inference System (FIS) is combined with a machine learning system consisting of five trained machine learning models that were trained in Experiment 2. The fuzzy system analyses the input (solar irradiation) and selects the trained machine learning model that would provide the most accurate model prediction of the PV string daily DC energy. 

**Figure 2:**
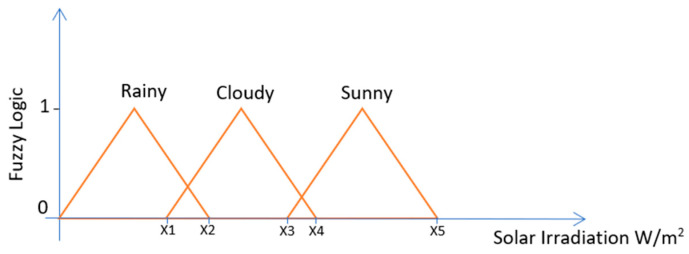
Illustration of a Fuzzy Logic System that associates ranges of solar irradiation values with weather conditions such as rainy, cloudy and sunny days.

**Figure 3:**
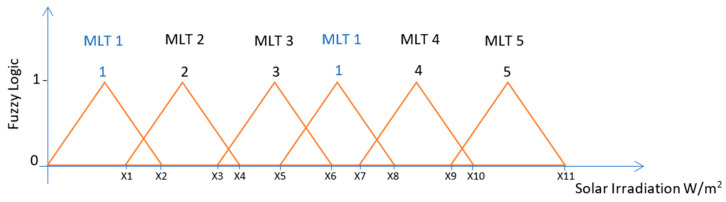
Illustration of a Takagi-Sugeno Fuzzy Logic System that categorises ranges of solar irradiation values for a machine learning technique.

There are many fuzzy systems such as the Mamdani fuzzy system that uses classes as the fuzzy logic output as illustrated in Figure 2 and the Takagi-Sugeno fuzzy system that uses a constant number as the fuzzy logic output as illustrated in Figure 3.

The fuzzy system used in this work is a single input and a single output Takagi-Sugeno fuzzy logic inference system. The output can only take 1 of 5 integer values that correspond to the MLT solution that is most appropriate as illustrated in Figure 3. Therefore, the regression tree is associated with the fuzzy output 1, the artificial neural network is associated with the fuzzy output 2, the multi-gene genetic programming is associated with the fuzzy output 3, the Gaussian process is associated with the fuzzy output 4, and finally the support vector machines for regression is associated with the fuzzy output 5. The advantage of this fuzzy system compared to the previous one illustrated in Figure 2, is that the same machine learning technique can be associated to different ranges of solar irradiation values, whereas in the previous fuzzy system the sunny day classification could only be used in a specific range of solar irradiation values.

The results of Experiment 2 provided information about the relationship between the solar irradiation values and the machine learning techniques that would provide the most accurate results, as presented in Table 2. It is possible to verify that certain daily solar irradiation ranges are associated to a certain machine learning technique that provides the best results, and therefore as an example there is the daily solar irradiation values between 4916 W/m^2^ and 5011 W/m^2^ is associated with the support vector machines for regression, and between 5597 W/m^2^ and 5761 W/m^2^ is associated with the regression tree. This information was used to implement the fuzzy system by grouping all the daily solar irradiation values into ranges (fuzzy input) and associating each of the ranges to a machine learning technique (fuzzy output).

**Figure 4:**
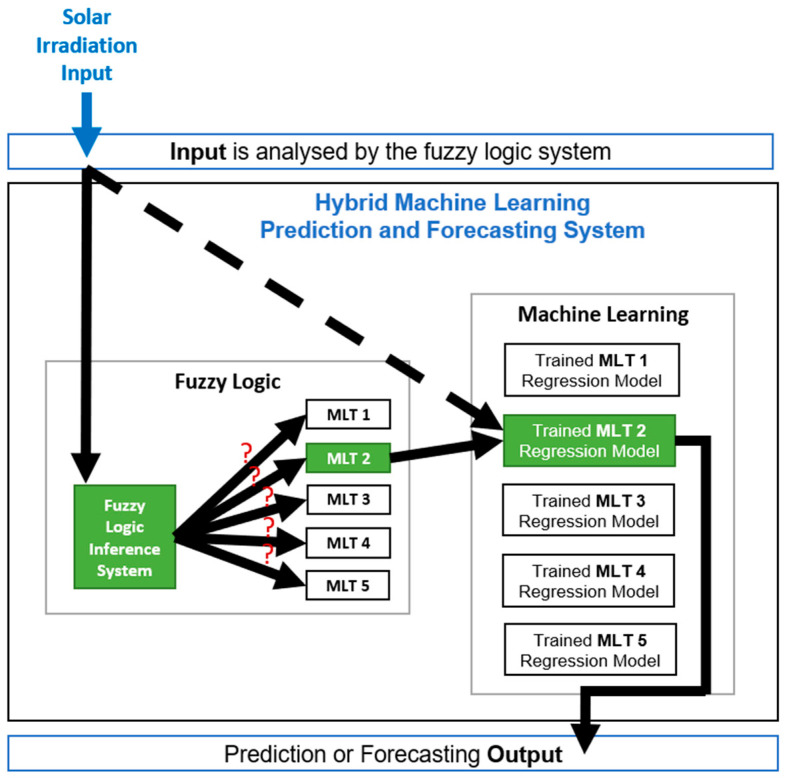
Hybrid Machine Learning Prediction and Forecasting System.

Figure 4 illustrates how the machine learning hybrid methodology system works, where the five trained models obtained from Experiment 2 are used together with a fuzzy system. The solar irradiation input is given to the hybrid system which then sends it to the fuzzy system to analyze and associate it with the machine learning technique that will provide the most accurate results based on the information obtained in Experiment 2. Knowing which machine learning technique will provide the most accurate results, the hybrid system sends the solar irradiation input to the trained machine learning model which then provides the prediction output of the PV string DC energy production.

**Figure 5:**
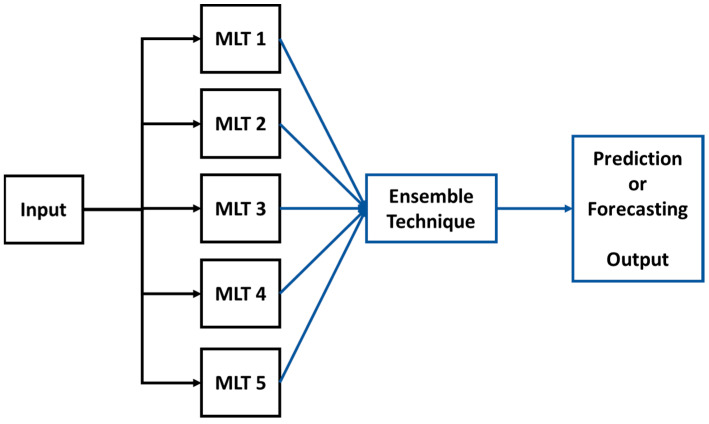
Diagram of the Ensemble Methodology that uses five different machine learning techniques (MLTs).

Experiment 4 represents the machine learning ensemble methodology described in the introduction Section, which is implemented by averaging the values provided by the five trained machine learning models obtained from Experiment 2. Figure 5 illustrates how the machine learning ensemble methodology system works, where the solar irradiation input is given to all five machine learning models. All of the output values of each of the five machine learning techniques are averaged, and this averaged value is the output provided by the machine learning ensemble methodology system.

A fifth experiment (Experiment 5) was conducted to resolve the issues raised by the poor results of the artificial neural networks. As a result, the configuration of the ensemble methodology system of Experiment 5 is exactly the same as the one presented in Experiment 4 with the exception that instead of the ensemble technique averaging all five machine learning techniques it would only average the top 3 best results given by the five machine learning models.

After analyzing the results from Experiment 4 and 5, a sixth experiment (Experiment 6) was conducted to verify the results of an ensemble methodology system that does not consider the results of the popular machine learning techniques such as the artificial neural networks and support vector machines. Therefore, Experiment 6 is an ensemble methodology system that considers only the regression tree, multi-gene genetic programming and Gaussian process model results for the averaging.

After determining which methodology provides the most accurate machine learning regression model outputs of the PV string energy production, the PV string fault detection method can take place, which includes the analysis of the deviation between the measured data and the prediction data. The presence of deviation between these two data values indicates the presence of a PV string fault. 

### 2.1. Hyperparameters of the Machine Learning Techniques

The following subsections briefly explain the commands and parameter settings of each of the machine learning models.

#### 2.1.1. Regression Tree 

The ‘fitrtree’ command returns a regression binary tree based on the input variables x and target variables y, where each branching nodes is split on the basis of the values of a column of inputs. The rest of the settings used were the default settings.

#### 2.1.2. Artificial Neural Networks

The train command returns a net model based on the parameter settings that follow. The parameters chosen for the ANN in this work include a multilayered feedforward backpropagation network using the Levenberg–Marquardt training algorithm, 10 neurons, 5000 epoch iterations, and the ‘mapminmax’ normalization for the inputs and the targets. The multilayered transfer function used in this work was the tan-sigmoid and linear purelin. Every time an artificial neural network model is trained it provides different outputs and this is because the initial weights of the neural connections are selected randomly by default and therefore these types of models are referred to as stochastic models. In order to minimize the issues related to this stochastic nature of the artificial neural network models, 10 of these models are trained and the best one is used in the experiments.

#### 2.1.3. Multi-Gene Genetic Programming 

The model is trained by using the ‘rungp’ training command and then it is tested by using the ‘mymodel’ command. The default control parameters to stop the tree evolution were set to population size of 250, timeout to 10 seconds, runs to 3, and max number of genes to 6. Since the multi-gene genetic programming models are stochastic models, just as the artificial neural network models, the same procedure of selecting the best model out of 10 was also adopted here.

#### 2.1.4. Gaussian Process 

The ‘fitrgp’ command returns a GPR model for inputs and continuous targets vector. In this work, the default kernel functions (squared exponential) and parameters (sigma) were used.

#### 2.1.5. Support Vector Machines for Regression 

The ‘fitrsvm’ command returns a full, SVM regression model trained using the input values in the matrix and the target values in the vector. The parameters chosen for the SVR include an epsilon value of 0.09, and the input data are standardized. The kernel used in this work for the SVR model was the default linear kernel.

## 3. Results

All the results of the six experiments are presented and discussed in this Section. Experiments 1 and 2 are used to determine the amount and type of data to include in the training dataset and also to determine which machine learning techniques provide the most accurate machine learning regression prediction outputs of the PV string DC energy.

Table 3 presents the results of Experiment 1 and shows that the Gaussian process machine learning technique provides the best results compared to the other four machine learning techniques in all of the scenarios while the ANN machine learning technique provides the worst results in all the scenarios. 

Table 4, shows that the Gaussian process machine learning technique provides the best results for scenarios 7, 8, 10, and 12. While the ANN machine learning technique provides the best results for scenario 9, the SVR machine learning technique provides the best results for scenario 11, and the multi-gene genetic programming machine learning technique provides the best results for scenario 13. 

The second-best results are presented in grey in Table 3 and Table 4. 

Overall, the results presented by Experiment 2 re lower than the ones presented in Experiment 1, even though Experiment 2 only uses 93 data samples for training.

Overall, experiments 1 and 2 suggest that the Gaussian process machine learning technique should be used to predict the daily DC solar energy outputs of the ODD and EVEN string of years before and after the ones used for training. The multi-gene genetic programming machine learning technique could also be considered, however this machine learning technique uses many more computational resources than the Gaussian process machine learning technique.

The Gaussian process technique provides the best results for all the ODD string scenarios in Experiment 2 (Table 4). This is expected since the machine learning techniques were trained only with the ODD strings of the year 2015, and even so, the Gaussian process technique provided the best results for the EVEN strings of scenario 8 and very good results for the EVEN string scenarios 11 and 13. 

The SVR machine learning technique provided the worst results in scenario 9 compared to all the other machine learning techniques.

**Figure 6:**
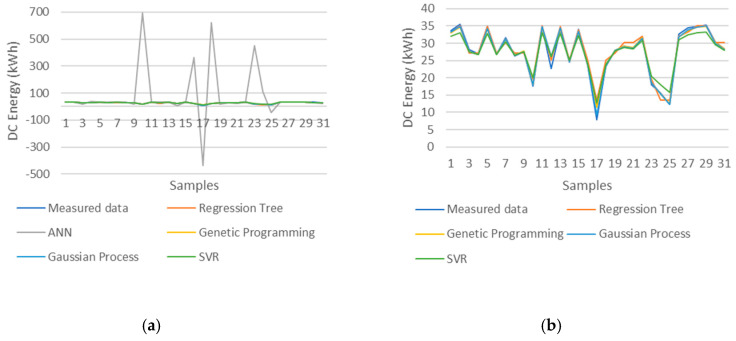
Predictions of PV string 1 in 2014 (**a**) All five machine learning techniques (**b**) All machine learning techniques except for ANN

Scenario 9 presents very interesting information about the behavior of the different machine learning techniques since the ANN machine learning technique provided the best results for this scenario, whereas it provides the worst results in all other scenarios, and its use cannot be considered in any case other than in this scenario because the NRMSE is higher than 5%. Figure 6 illustrates the machine learning model output predictions of the 2014 PV string 1 DC energy. It is possible to observe why the ANN error values (RMSE and NRMSE) present such bad results, since it is easy to see the peaks of the ANN predictions which indicate that this machine learning techniques has difficulty in accurately predicting certain PV string DC energy values compared to the other four machine learning techniques. This information gained from comparing Experiments 1 and 2 indicates that the ANN machine learning technique requires at least the two groups of PV string historical data (ODD and EVEN) with 186 samples to provide better results such as the ones provided in Experiment 1 (Table 3). This information also indicates that, if more data are included in the training dataset of the ANN machine learning technique, it would provide even better results, and therefore the ANNs require many more training data samples than any of the other four machine learning techniques do. The results of scenario 9 of Experiment 2 indicate that the ANN machine learning technique deals well with familiar data, since the machine learning techniques were trained with the July ODD strings of the year 2015 and tested with the July EVEN strings of the same year. Therefore, the ANN is the MLT that is the most sensitive to the size of the training dataset as well as the type (familiar) of data in the training dataset.

The SVR provides acceptable model results, however they are higher than the less popular machine learning techniques (regression tree, multi-gene genetic programming and Gaussian process). For the SVR more samples in the training dataset is not the answer since the model values do not vary much between Experiment 1 (more samples in training dataset) and Experiment 2 (less samples in the training dataset). The less popular machine learning techniques do not need as much data as the ANN to provide very accurate model results. Therefore, even though the ANN and SVR MLTs are the most popular (used the most by the research community) it does not mean that they are the ones that are going to provide the best results. Experiments 1 and 2 show that it is exactly the two most popular MLTs (ANN and SVR) that provide the worst results.

Experiment 3 is also presented in Table 4 in order to simplify the visual comparison between the results of Experiments 2 and 3. Experiment 3 presents the results obtained from the machine learning hybrid methodology system which combines the use of a fuzzy logic system and all five trained models obtained from Experiment 2. The Takagi-Sugeno type fuzzy logic inference system is used to select one of the five machine learning techniques that would provide the most accurate prediction by analyzing the solar irradiation input that is given. The error results of Experiment 3 are much lower compared to the error results of Experiments 1 and 2, and therefore the machine learning hybrid methodology system provides much better results than the mono systems used in Experiments 1 and 2 where the idea is to rely on only one machine learning technique to provide the best results. 

Experiments 1, 2, and 3 clearly show that different machine learning techniques provide good results for different types of data in different scenarios, and therefore different machine learning techniques deal differently with different data. These experiments prove that it is not possible to select only one machine learning technique to provide the best results for all types of data in all types of scenarios, and therefore the best solution is to use various machine learning techniques in a hybrid system that will select each one to be used to their best ability based on the analysis that is made to the input that is given to the hybrid system as described in the methodology Section.

The results of Experiment 3 are better than the ones from Experiments 1 and 2, and therefore are repeated in Table 5 to simplify the visual comparison between the results of Experiments 3, 4, 5, and 6. 

Experiments 4, 5, and 6 all use the ensemble methodology system to provide the prediction values of the PV string DC energy, however each one of them has a different configuration.

Experiments 4 and 5 use the ensemble methodology which involves using a simple ensemble technique that averages the results provided by the five machine learning techniques. These two experiments differ from each other in the number of machine learning results they average. In Experiment 4, the ensemble technique averages all five machine learning technique results, while in Experiment 5 the ensemble technique only averages the top three results provided by the machine learning techniques. Overall, the results provided in Experiment 5 are better than the ones provided in Experiment 4. This is expected since the results from the artificial neural networks are not always very good compared to the results from the other machine learning techniques and therefore when it is used in an averaging ensemble technique the results are not going to be the best. Therefore, the methodology that selects the top three results to use in the ensemble methodology (Experiment 5) is better than using all five results from the five machine learning techniques as done in Experiment 4.

Experiment 6 uses the ensemble methodology described in Experiments 4 and 5, however instead of considering the averaging of all the results from the five machine learning techniques, Experiment 6 only considers the results of the less popular machine learning techniques such as the regression tree, multi-gene genetic programming, and the Gaussian process. Table 5 shows that the overall results of Experiment 6 are more accurate than the ones provided by Experiments 4 and 5, indicating that the popular machine learning techniques such as the artificial neural networks and the support vector machines negatively influence the final results of the ensemble methodology system.

Once again, the results of Experiment 3 are better than the ones of Experiments 4, 5 and 6, as expected since the results provided by Experiment 3 are precise compared to the results provided by Experiments 4, 5 and 6 which are average results. Therefore, Experiment 3 provides the best results out of all the experiments carried out in this research work, which means that the machine learning hybrid methodology is the one that provides the most accurate results of the four machine learning methodologies such as the data mining methodology, the machine learning technique benchmarking methodology, the hybrid methodology, and the ensemble methodology.

**Figure 7:**
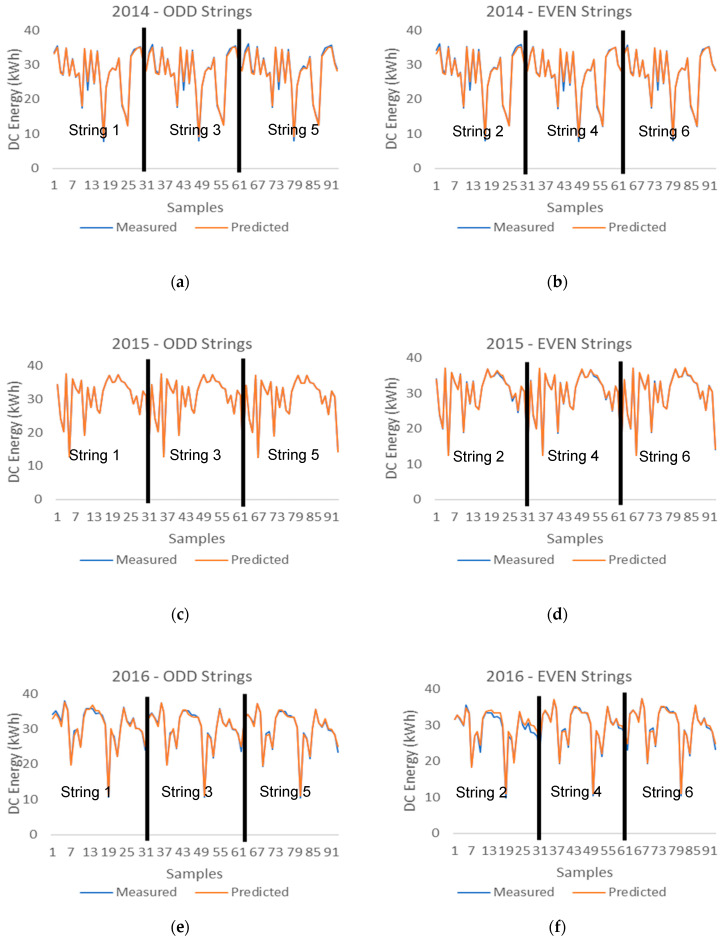

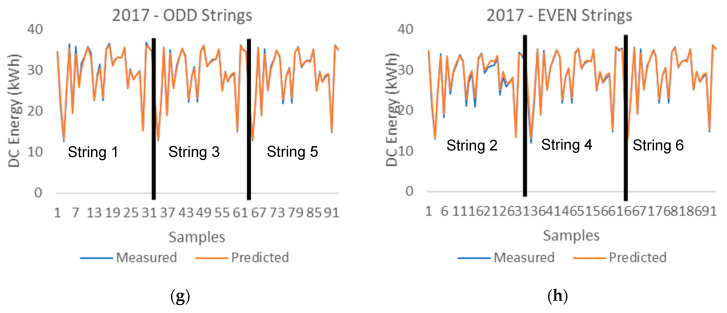
DC energy measured vs. predicted data provided by the hybrid system (Fuzzy + 5MLTs) (**a**) 2014 ODD PV Strings (**b**) 2014 EVEN PV Strings (**c**) 2015 ODD PV Strings (**d**) 2015 EVEN PV Strings (**e**) 2016 ODD PV Strings (**f**) 2016 EVEN PV Strings (**g**) 2017 ODD PV Strings (**h**) 2017 EVEN PV Strings.

Figure 7 shows how the DC energy measured data are closely correlated to the prediction data provided by the machine learning hybrid methodology system, which includes the combination of the fuzzy logic system and the machine learning system that consists of five trained machine learning models. 

**Figure 8:**
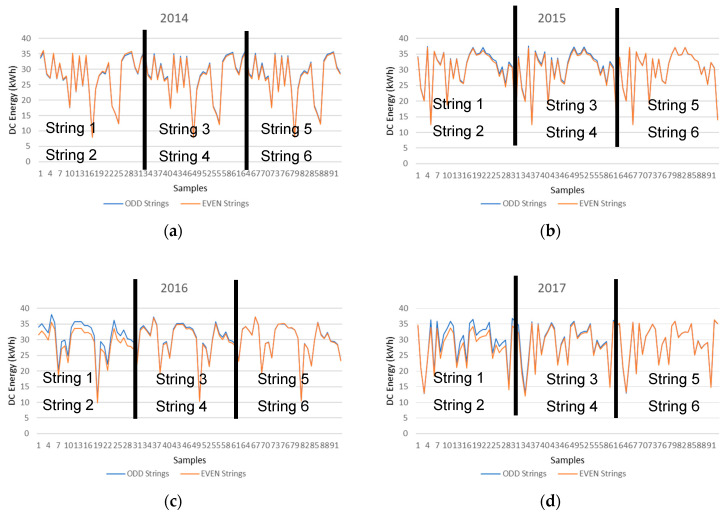
DC energy predictions of the ODD and EVEN PV strings provided by the machine learning hybrid methodology system (**a**) 2014 (**b**) 2015 (**c**) 2016 (**d**) 2017.

Once the machine learning methodology that provides the best machine learning regression model outputs of the PV string DC energy is determined, the next step is to analyze the deviation of the DC energy values of the ODD and EVEN PV strings. Figure 8 illustrates the prediction values of the DC energy values of the ODD and EVEN PV strings provided by the machine learning hybrid methodology system. This way, only the prediction values are considered, and the measured data are not, and therefore the deviation analysis is comparing the prediction values of the neighboring PV strings. 

The neighboring strings are Strings 1 and 2, Strings 3 and 4, and finally Strings 5 and 6. The x axis represents the number of samples and the y axis represents the DC energy of the PV Strings. All samples between 1 and 31 belong to Strings 1 (ODD) and 2 (EVEN), between 32 and 62 belong to Strings 3 (ODD) and 4 (EVEN), and finally all samples between 33 and 93 belong to Strings 5 (ODD) and 6 (EVEN).

The four graphs presented in Figure 8, illustrate the PV string DC energy production of the ODD and EVEN strings in the different years of July. When analyzing the results from the ODD and EVEN strings of the year 2014, it is verified that they are very similar to each other and therefore indicate that all strings are producing solar energy in a similar way. However, this production similarity does not take place in the years 2016 and 2017. It is clearly noticeable that the production in String 2 is lower compared to String 1 in years 2016 and 2017 when compared to the years 2014 and 2015, and therefore a deviation between the prediction values of the neighboring PV strings indicate that a PV system fault has been detected since this deviation does not take place in previous years. 

The reduction in the PV string production can be attributed to a number of reasons such as PV soiling, PV shading, cabling, PV module fault, or others. String 2 has a loss of approximately 2% compared to the past and this might be due to only one PV module in the PV string not producing at its maximum output and therefore dragging down the production of the whole PV string with it, due to the connection configuration of all PV modules in the string which are connected in series. The next step should be to manually inspect the string and each PV module individually to identify the anomaly and correct it. 

**Figure 9:**
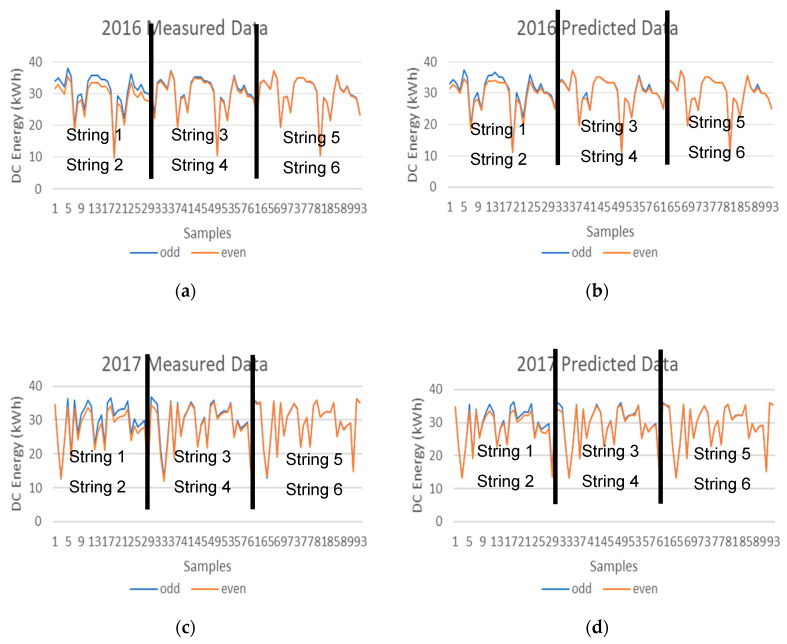
DC energy values of the ODD and EVEN PV strings (**a**) 2016 measured data (**b**) 2016 prediction data (**c**) 2017 measured data (**d**) 2017 prediction data.

The DC energy deviation analysis can also be performed by comparing the measured data of the neighboring PV strings as illustrated in Figure 9, however the deviation between the measured data (Figure 9a,c) is more visible than the deviation between the prediction data (Figure 9b,d).

## 4. Conclusions

The aim of this work was to determine which machine learning methodology, out of four, would provide the most accurate machine learning model predictions for using in PV string performance analysis and fault detection using a very small data sample. In conclusion, the aim of this work was achieved by using a new proposed machine learning hybrid methodology described in Experiment 3 to provide the most accurate machine learning regression model outputs of the daily DC energy of the PV strings. 

This hybrid methodology system combines the use of a Takagi-Sugeno type fuzzy logic inference system with the use of a machine learning system that has five trained machine learning models (regression tree, artificial neural network, multi-gene genetic programming, Gaussian process and support vector machines for regression) that were trained in Experiment 2. The fuzzy system analyses the solar irradiation input value given to the hybrid system and then selects the machine learning model that would provide the most accurate prediction results.

After determining which methodology provided the most accurate machine learning predictions, the PV string fault detection method was carried out by using the deviation analysis. The PV string fault detection was clearly identified in the deviation analysis when using the prediction values of the neighboring PV strings. As a result, this work also proposes a new method that is used to successfully detect PV string faults which includes the analysis of the deviation between the prediction values of each of the strings and not requiring the measured data to verify the presence of a PV string faults. The PV system fault is detected when the predictions of the ODD strings are deviated from the predictions of the EVEN strings. 

The benchmark analysis that was performed in Experiment 2 showed that the worst results were provided by the popular machine learning techniques (ANN and SVR), while the best results were provide by the less popular machine learning techniques (regression tree, multi-gene genetic programming and Gaussian process). The results obtained from Experiment 2 provided information about the behavior of the five machine learning techniques when dealing with different types of data from different scenarios. This information lead to the conclusion that it is not possible to select only one machine learning technique to deal well with all types of data in all types of scenarios. Therefore, the solution would be to use all of the five machine learning techniques in a hybrid system (Experiment 3) and have a separate system (Fuzzy system) to select one of the five models to provide the most accurate prediction results. This way, each machine learning technique is used based on their ability to accurately predict the DC energy output of the PV strings. It should be noted that all the conclusions obtained about the generalization of the different machine leaning models are mostly driven by the small size of the training sample dataset (93 samples).

A new ensemble methodology is also proposed in this work and is presented in Experiments 4, 5, and 6 where the five different machine learning techniques are used in the ensemble methodology system to provide the prediction results of the DC energy of the PV strings. However, the results provided by the hybrid system in Experiment 3 were much better than the average ones provided by the ensemble system in Experiments 4, 5, and 6.

## Figures and Tables

**Table 1:** Machine learning tribes and respective master algorithms [11].

	Tribe	Master Algorithm	Machine Learning Techniques Selected for This Work
**Tribe 1**	Symbolists	Inverse deduction/Induction	Regression Tree
**Tribe 2**	Connectionists	Backpropagation	Artificial Neural Network
**Tribe 3**	Evolutionaries	Genetic Programming	Multi-Gene Genetic Programming
**Tribe 4**	Bayesians	Probabilistic Inference	Gaussian Process
**Tribe 5**	Analogizers	Kernel Machines	Support Vector Machines

**Table 2:** Samples from 2014 and corresponding best machine learning techniques (MLTs).

Date	Measured			Estimated Output				
	Input Solar Irradiation (W/m^2^)	Output Solar Energy (kWh)	Regression Tree	ANN	Multi-Gene Genetic Programming	Gaussian Process	SVR	Best MLT
18/07/2014	4916	23.7	25.1	620.9	23.3	23.3	23.9	SVR
16/07/2014	5011	24.0	25.1	364.0	23.7	23.7	24.2	SVR
12/07/2014	5597	22.6	25.1	31.4	26.1	26.1	26.2	Regression Tree
04/07/2014	5747	27.0	27.1	37.2	26.7	26.7	26.7	Regression Tree
06/07/2014	5761	27.1	27.1	33.2	26.7	26.7	26.8	Regression Tree
03/07/2014	5976	28.3	27.1	15.3	27.6	27.6	27.5	Genetic Programming
09/07/2014	5993	27.9	27.1	16.0	27.7	27.6	27.6	Genetic Programming
19/07/2014	6074	28.0	27.1	19.0	28.0	27.9	27.8	Genetic Programming
31/07/2014	6146	28.5	30.1	21.3	28.3	28.2	28.1	Genetic Programming
20/07/2014	6339	29.1	30.1	25.9	29.1	29.0	28.7	Genetic Programming
30/07/2014	6610	30.4	30.1	29.7	30.3	30.1	29.7	Genetic Programming
07/07/2014	6862	31.7	30.1	31.6	31.3	31.2	30.5	ANN
22/07/2014	6936	32.1	32.0	32.0	31.6	31.5	30.8	ANN
26/07/2014	7004	32.6	32.0	32.3	31.9	31.8	31.0	ANN
01/07/2014	7304	34.0	33.0	33.2	33.1	33.2	32.0	Gaussian Process
27/07/2014	7434	34.4	33.3	33.4	33.7	33.9	32.5	Gaussian Process

**Table 3:** Experiment 1 - Train ALL 6 strings from 2015 - Train with 186 samples.

Dataset Year			July 2014		July 2016		July 2017	
Scenarios			Scenario 1	Scenario 2	Scenario 3	Scenario 4	Scenario 5	Scenario 6
Tribe	MLT	Error Analysis	ODD Strings	EVEN Strings	ODD Strings	EVEN Strings	ODD Strings	EVEN Strings
**1**	**Regression** **Tree**	**RMSE** **kWh**	1.266	1.256	1.066	1.398	1.051	1.303
		**NRMSE**	3.248%	3.222%	2.733%	3.586%	2.695%	3.343%
**2**	**ANN**	**RMSE** **kWh**	4.068	3.356	2.245	2.202	1.905	5.273
		**NRMSE**	10.431%	8.607%	5.756%	5.646%	4.887%	13.522%
**3**	**Multi-Gene Genetic Programming**	**RMSE** **kWh**	2.057	1.995	0.862	1.383	0.640	1.104
		**NRMSE**	5.275%	5.116%	2.210%	3.546%	1.643%	2.832%
**4**	**Gaussian Process**	**RMSE** **kWh**	0.889	0.842	0.853	1.326	0.629	1.100
		**NRMSE**	2.281%	2.158%	2.189%	3.400%	1.614%	2.821%
**5**	**SVR**	**RMSE** **kWh**	1.874	1.864	1.478	1.397	1.616	1.561
		**NRMSE**	4.807%	4.779%	3.791%	3.583%	4.144%	4.002%

**Table 4:** Experiment 2 and Experiment 3 - Train 3 strings from 2015 - Train with 93 samples.

Data Set Year Scenarios			July 2014		July 2015	July 2016		July 2017	
			Scenario 7	Scenario 8	Scenario 9	Scenario 10	Scenario 11	Scenario 12	Scenario 13
Tribe	MLT	Error Analysis	ODD Strings	EVEN Strings	EVEN Strings	ODD Strings	EVEN Strings	ODD Strings	EVEN Strings
**1**	**Regression** **Tree**	**RMSE** **kWh**	1.489	1.532	0.649	1.144	1.605	1.331	1.713
		**NRMSE**	3.818%	3.929%	1.664%	2.935%	4.115%	3.413%	4.394%
**2**	**ANN**	**RMSE** **kWh**	206.84	183.48	0.567	114.964	114.996	208.54	208.965
		**NRMSE**	530%	470%	1.455%	295%	295%	535%	536%
**3**	**Multi-Gene Genetic Programming**	**RMSE** **kWh**	1.024	1.091	0.728	0.873	1.546	0.624	1.203
		**NRMSE**	2.628%	2.798%	1.868%	2.238%	3.964%	1.599%	3.084%
**4**	**Gaussian Process**	**RMSE** **kWh**	0.826	0.816	0.735	0.850	1.436	0.616	1.211
		**NRMSE**	2.118%	2.092%	1.884%	2.179%	3.682%	1.579%	3.104%
**5**	**SVR**	**RMSE** **kWh**	1.904	1.899	1.637	1.489	1.427	1.638	1.599
		**NRMSE**	4.883%	4.869%	4.199%	3.819%	3.659%	4.199%	4.099%
	**Experiment 3–Fuzzy Logic with Experiment 2 prediction Model Results**								
			**Scenario 14**	**Scenario 15**	**Scenario 16**	**Scenario 17**	**Scenario 18**	**Scenario 19**	**Scenario 20**
**Fuzzy + 5 MLTs**		**RMSE** **kWh**	0.614	0.627	0.232	0.516	0.746	0.502	0.722
		**NRMSE**	1.574%	1.607%	0.596%	1.324%	1.912%	1.288%	1.850%

**Table 5:** Experiments 3, 4 and 5 - Train 3 strings from 2015 - Train with 93 samples.

Data Set Year			July 2014		July 2015	July 2016		July 2017	
		Error Analysis	ODD Strings	EVEN Strings	EVEN Strings	ODD Strings	EVEN Strings	ODD Strings	EVEN Strings
**Experiment 3–Fuzzy Logic with Experiment 2 prediction Models **									
**Fuzzy + 5 MLTs**			Scenario 14	Scenario 15	Scenario 16	Scenario 17	Scenario 18	Scenario 19	Scenario 20
		**RMSE** **kWh**	0.614	0.627	0.232	0.516	0.746	0.502	0.722
		**NRMSE**	1.574%	1.607%	0.596%	1.324%	1.912%	1.288%	1.850%
**Experiment 4–Ensemble Methodology with Experiment 2 prediction Model**									
**Ensemble** **ALL** **5 MLTs**			Scenario 21	Scenario 22	Scenario 23	Scenario 24	Scenario 25	Scenario 26	Scenario 27
		**RMSE** **kWh**	41.536	36.585	0.624	22.777	22.899	42.143	42.405
		**NRMSE**	106.50%	93.81%	1.601%	58.404%	58.716%	108.06%	108.73%
**Experiment 5–Ensemble Methodology with Experiment 2 prediction Models**									
**Ensemble** **Top 3**			Scenario 28	Scenario 29	Scenario 30	Scenario 31	Scenario 32	Scenario 33	Scenario 34
		**RMSE** **kWh**	2.142	2.048	0.643	1.641	1.484	1.122	1.109
		**NRMSE**	5.493%	5.251%	1.648%	4.208%	3.806%	2.878%	2.845%
**Experiment 6–Ensemble Methodology with Experiment 2 prediction Models**									
**Ensemble** **ALL without ANN and SVR**			Scenario 35	Scenario 36	Scenario 37	Scenario 38	Scenario 39	Scenario 40	Scenario 41
		**RMSE** **kWh**	1.017	1.057	0.647	0.865	1.422	0.801	1.316
		**NRMSE**	2.607%	2.710%	1.660%	2.217%	3.645%	2.054%	3.375%
